# Human papillomavirus 16–positive supraclavicular cutaneous squamous cell carcinoma metastatic to the level IV supraclavicular lymph nodes

**DOI:** 10.1016/j.jdcr.2020.07.002

**Published:** 2020-07-11

**Authors:** Hannah Dekker, Rolf J. Bun, Doriene C. Mulder, Nelly Breeuwsma, Jasper I. van der Rhee, Núria Guimerà, Wim Quint, Maarten H. Vermeer, Jan N. Bouwes Bavinck

**Affiliations:** a Department of Oral and Maxillofacial Surgery, Noord-West Ziekenhuisgroep, Alkmaar, Netherlands; b Department of Pathology, Noord-West Ziekenhuisgroep, Alkmaar, Netherlands; c Department of Dermatology, Noord-West Ziekenhuisgroep, Alkmaar, Netherlands; d DDL Diagnostic Laboratory, Research and Development, Rijswijk, Netherlands; e Department of Dermatology, Leiden University Medical Center, Leiden, Netherlands

**Keywords:** cSCC, cutaneous squamous cell carcinoma, head and neck squamous cell carcinoma, HNSCC, human papillomavirus, HPV, human papillomavirus, SCC, squamous cell carcinoma

## Introduction

The role of (high-risk) human papillomaviruses (HPVs) in cervical and anogenital squamous cell carcinomas (SCCs), as well as a subset of oropharyngeal cancers, is well recognized. The majority (>90%) of the HPV-associated oropharyngeal SCCs are caused by HPV 16.^1^ The association between HPV and cutaneous SCC remains to be elucidated and is thought to involve cutaneous types of HPV as opposed to mucosal types such as HPV 16.^2^ In this report, we describe the presence of HPV 16–positive cervical lymph node metastases in a patient with a previous cutaneous SCC and no evidence of an oropharyngeal malignancy.

## Case report

A 76-year old man presented to our oral and maxillofacial surgery department with a left-sided supraclavicular mass. Eight months earlier, his general practitioner had removed a 1.8-cm, exophytic, growing, cutaneous SCC located superior to the left clavicle, approximately 2 cm lateral to the sternoclavicular joint. The excision performed by the general practitioner was not radical, and subsequently the 4-cm scar was re-excised with a wide local excision and sutured. The histology showed a 9.0-mm-thick, poorly differentiated cutaneous SCC without perineural or lymphovascular invasion and was classified as a T2 tumor according to the American Joint Committee on Cancer, tumor-node-metastasis staging system, 7th edition. The patient had diabetes mellitus type 2, hypertension, renal impairment, and coronary artery disease. Two years earlier, he had had a hemicolectomy because of a T4N0 cecum carcinoma. Except for actinic keratoses, he had no (pre) skin cancer history.

Fluor-18-deoxyglucose positron emission tomography–computed tomography showed bilateral supraclavicular FDG-avid masses. An ultrasonographically guided fine-needle aspiration cytology punctate was taken from these masses, histopathology assessment of the aspirate showed SCC cells. The cells were routinely screened for HPV: DNA isolation (prot.K-SDS method), polymerase chain reaction, and HPV testing for high-risk HPV types 16, 18, 45, 31, 33, 52, 58, 35, 59, 56, 51, 39, 68, 73, 82, 53, 66, and 70 and low-risk HPV types 6, 11, 40, 42, 43, and 44/55 (PapilloCheck and PapilloCheck high risk; Greiner, Frickenhausen, Germany).

A selective neck dissection was planned because of a diagnosis of cervical lymph node metastasis of the cutaneous SCC; however, the HPV screening result of the punctate was positive for HPV 16. Surgery was cancelled because HPV 16 positivity is suspicious for a primary oropharyngeal tumor. The patient underwent panendoscopy, bilateral tonsillectomy, and tongue base and nasopharyngeal biopsies, which showed no malignancy.

The tumor specimen of the supraclavicular cutaneous SCC was then screened for HPV by the aforementioned method and also was positive for HPV 16. Subsequently, the patient underwent a bilateral selective neck node dissection (levels III-V left and level V right). In level IV left and V right, metastatic nodes with extensive extranodal growth into the resection margin were found. The final pathologic diagnosis was HPV 16–positive cutaneous SCC located in the head and neck area, with bilateral supraclavicular lymph node metastasis. The patient was treated with adjuvant radiotherapy. Soon after finishing his treatment, he presented with pulmonary, mediastinal, and calvarial bone metastasis. He died of disseminated disease 13 months after initial presentation.

## Discussion

**Fig 1:**
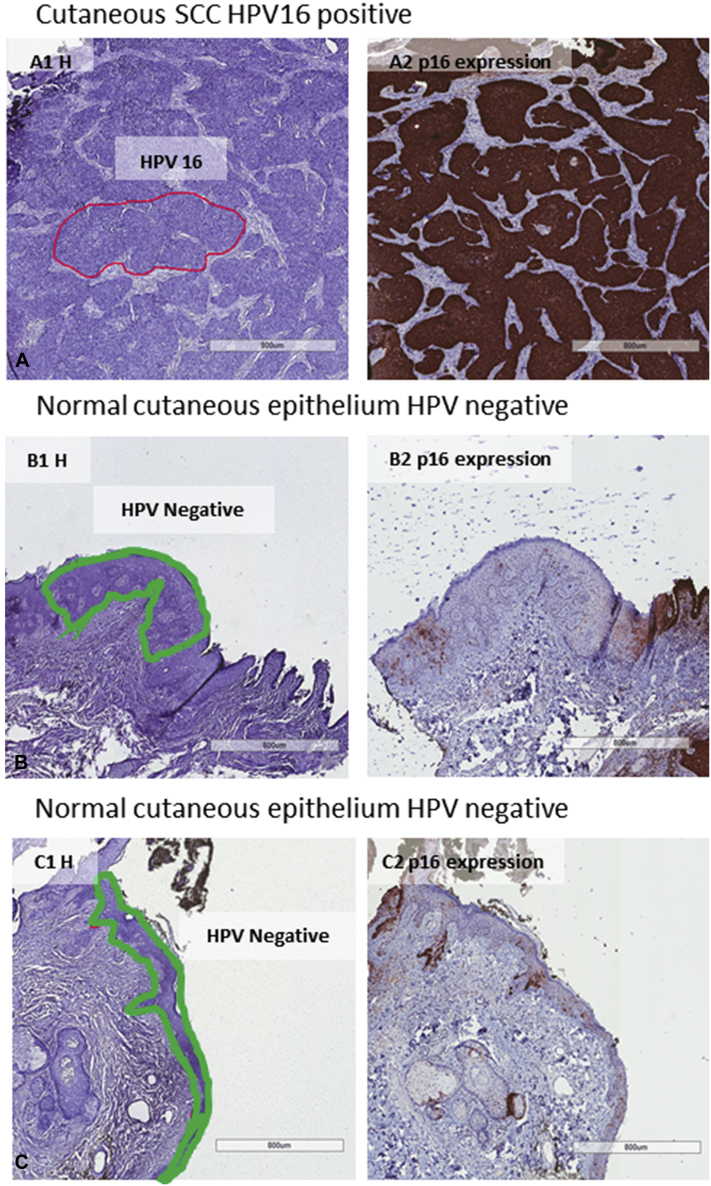
Histologic images from cutaneous squamous cell carcinoma (**A**) and normal cutaneous epithelium adjacent to the cutaneous squamous cell carcinoma (**B**, **C**). Human papillomavirus 16 was localized by laser-capture microdissection polymerase chain reaction in the cutaneous squamous cell carcinoma, but not in normal epithelium. p16 (Brown areas) was expressed only in the cutaneous squamous cell carcinoma and not in normal epithelium. *HPV*, Human papillomavirus; *SCC*, squamous cell carcinoma.

**Fig 2:**
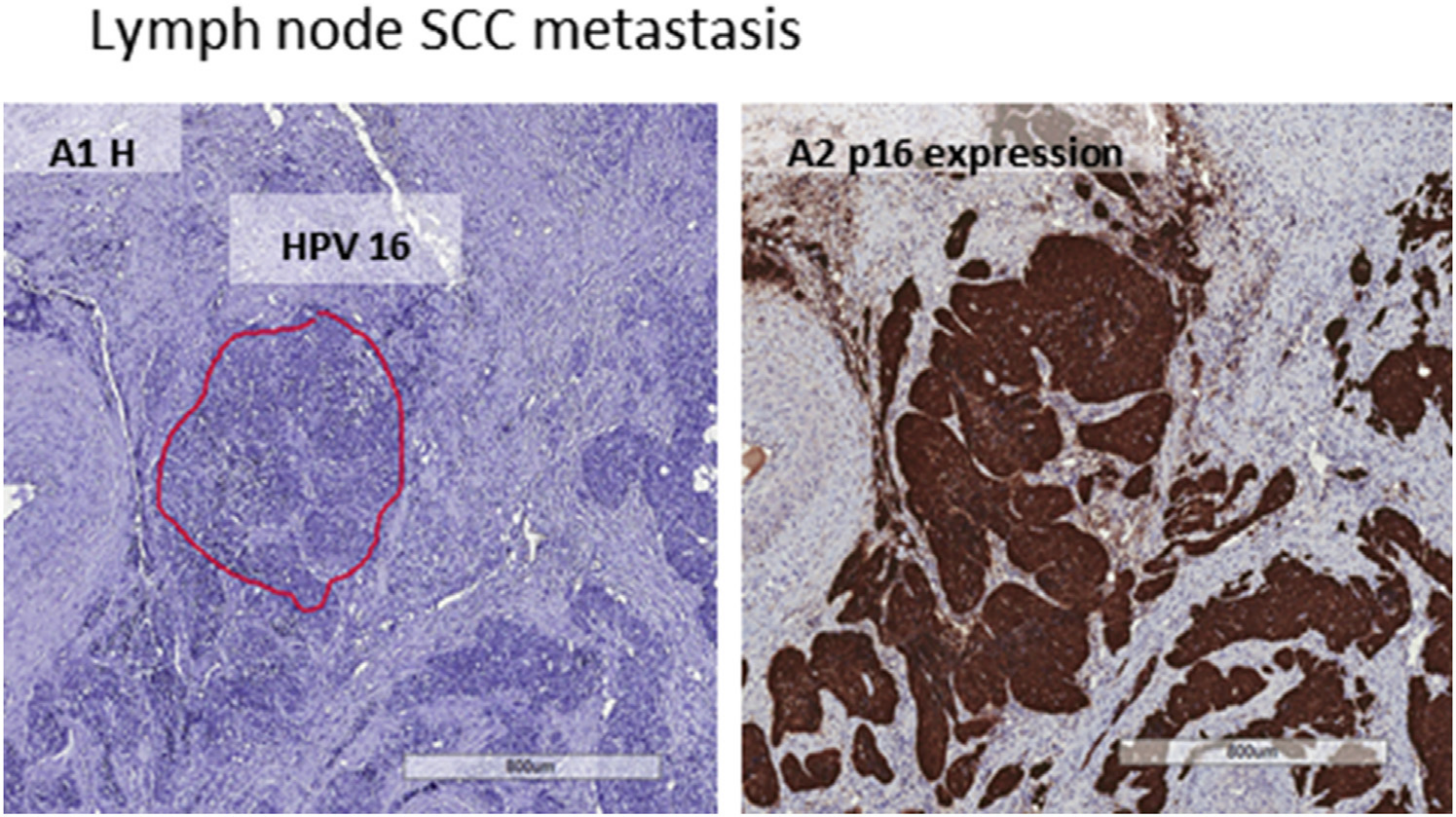
Histologic image from lymph node squamous cell carcinoma metastasis. Human papillomavirus 16 was localized by laser-capture microdissection polymerase chain reaction in the squamous cell carcinoma metastasis and p16 (brown area) was also expressed in the tumor cells. *HPV*, Human papillomavirus.

The detection of HPV 16 in this tumor was an unexpected finding because it is not known to be associated with cutaneous SCC in this anatomic region. HPV 16 detection in the tumor cells and in the metastasis was confirmed by laser-capture microdissection and the SPF_10_-LiPA_25_ system (Labo Bio-Medical Products, Rijswijk, the Netherlands).^3^ The presence of cutaneous HPVs was analyzed by beta HPV test.^4^ Laser-capture microdissection polymerase chain reaction is a very sensitive and specific technique to histologically localize HPV in heterogenous formalin fixed paraffin embedded whole-tissue sections. HPV 16 was specifically localized in tumor cells and in the metastasis, suggesting a carcinogenic role in these malignancies. Beta HPV 23 was detected (weakly) in the skin tumor, but not in the lymph node metastasis. p16^INK4a^ Biomarker, a sensitive but not specific marker of oncogenic HPV-E7 gene activity, was analyzed by immunohistochemistry.^3^ p16 Was overexpressed in the skin tumor and in the lymph node metastasis (Figs 1 and 2). p16 Overexpression might also be induced by ultraviolet radiation; therefore, it is not a conclusive result to determine the etiologic role of HPV in this tumor.

High-risk types of HPV cause almost 5% of all cancers worldwide and are mainly associated with cervical, anogenital, and oropharyngeal cancer.^3^ Outside the genital and oropharyngeal regions, the high-risk HPV-associated tumors (mainly HPV 16) are rare and reported cases are usually localized on the hands and feet, in particular the nailbed.^5^

The cutaneous HPV types are widely present in the skin of healthy individuals.^2^ The association between beta HPV and cutaneous SCC is still unclear; however, there is accumulating evidence that beta HPVs act as a cocarcinogen because they can synergize with ultraviolet exposure in carcinogenesis.^6^

Approximately 60% of oropharyngeal cancers in the United States and 30% in Europe are HPV 16 positive. The majority (>90%) of the HPV-associated oropharyngeal SCCs are caused by HPV 16.^1^ Routine high-risk-type HPV testing is therefore standard of care in the evaluation of patients with oropharyngeal cancer and of patients with cervical lymph node metastasis of SCC with an unknown primary cause.^7^

HPV 16 is generally not considered to play a role in the carcinogenesis of cutaneous SCC. The incidence of HPV 16–positive cutaneous SCC is unknown and has not often been described in the literature. Multiple studies using DNA in situ hybridization to detect high-risk HPV types in cutaneous SCCs located in the head and neck region failed to detect positive samples.^8^

Asgari et al^9^ used polymerase chain reaction to detect various HPV types in cutaneous SCCs (85 samples, digits excluded) and 2 were HPV 16 positive. Andersson et al^10^ screened serum samples from cutaneous SCC patients for HPV 16 and 18 and used polymerase chain reaction for the tumor blocks from seropositive patients. Of 81 tumors, only 4 were positive for HPV 16 and none for HPV 18. Tumor locations were perineal, limb, and lip.

When HPV 16 is detected in whole-tissue sections, a very sensitive and specific methodology for HPV DNA detection, such as the laser-capture microdissection–polymerase chain reaction SPF_10_-LiPA_25_ system, should be used to exclude potential contamination from adjacent tissue.^3^ In this Case Report, we have confirmed the presence of HPV 16 in tumor cells from a primary cutaneous SCC and its related neck lymph node metastasis. The strong expression of p16^INK4a^ in this cutaneous SCC might have been related to HPV 16 E7 oncoprotein activity or have been induced by ultraviolet radiation. In accordance with our findings, we believe that HPV 16 was the major reason for the oncogenesis in this tumor. HPV 23 seems more of a bystander than a participant in this case.

In conclusion, this case illustrates that test results for cutaneous SCCs can be positive for HPV 16. In HPV 16–positive lymph node metastasis from an unknown primary cause, a cutaneous SCC (or history of one) should be considered as a possible primary tumor site. When available, histologic samples of a cutaneous SCC should be tested for HPV 16.

## References

[1] Marur S., D'Souza G., Westra W.H., Forastiere A.A. (2010). HPV-associated head and neck cancer: a virus-related cancer epidemic. Lancet Oncol.

[2] Hasche D., Vinzon S.E., Rosl F. (2018). Cutaneous papillomaviruses and non-melanoma skin cancer: causal agents or innocent bystanders?. Front Microbiol.

[3] Guimerà N., Lloveras B., Lindeman J. (2013). The occasional role of low-risk human papillomaviruses 6, 11, 42, 44, and 70 in anogenital carcinoma defined by laser capture microdissection/PCR methodology: results from a global study. Am J Surg Pathol.

[4] De Koning M., Quint W., Struijk L. (2006). Evaluation of a novel highly sensitive, broad-spectrum PCR-reverse hybridization assay for detection and identification of beta-papillomavirus DNA. J Clin Microbiol.

[5] Riddel C., Rashid R., Thomas V. (2011). Ungual and periungual human papillomavirus-associated squamous cell carcinoma: a review. J Am Acad Dermatol.

[6] Bouwes Bavinck J.N., Feltkamp M.C.W., Green A.C. (2018). Human papillomavirus and posttransplantation cutaneous squamous cell carcinoma: a multicenter, prospective cohort study. Am J Transplant.

[7] Lewis J.S., Beadle B., Bishop J.A. (2018). Human papillomavirus testing in head and neck carcinomas: guideline from the College of American Pathologists. Arch Pathol Lab Med.

[8] Satgunaseelan L., Chia N., Suh H. (2017). p16 Expression in cutaneous squamous cell carcinoma of the head and neck is not associated with integration of high risk HPV DNA or prognosis. Pathology.

[9] Asgari M.M., Kiviat N.B., Critchlow C.W. (2008). Detection of human papillomavirus DNA in cutaneous squamous cell carcinoma among immunocompetent individuals. J Invest Dermatol.

[10] Andersson K., Luostarinen T., Strand A.S. (2013). Prospective study of genital human papillomaviruses and nonmelanoma skin cancer. Int J Cancer.

